# In this month’s Bulletin

**DOI:** 10.2471/BLT.18.001018

**Published:** 2018-10-01

In the editorial section, Ivo Kocur et al. (666) introduce this theme issue on the future of eye care in a changing world.

Karin Weyer et al. (667) discuss the improvements needed in treatment regimens for drug-resistant tuberculosis.

In the news section, Jack Latimore (670–671) reports on efforts to address eye-care needs of Australia’s Indigenous people. In an interview, Haroon Awan explains to Fiona Fleck (672–673) how several low- and middle-income countries are incorporating eye care into their national health strategies.

## Brazil, China, India, Islamic Republic of Iran, Mexico, Nepal, Nigeria, Peru, South Africa, Thailand, Timor-Leste, United Republic of Tanzania, Viet Nam

### Providing eye-care to school children

Anthea M Burnett et al. (682–694) gather the evidence on school-based eye-care services.

## India

### Including eyesight in public health interventions

Venkata SM Gudlavalleti et al. (705–715) describe how avoidable blindness is handled.

## Peru

### Getting people to specialists

Omar Salamanca et al. (674–681) implement a diabetic retinopathy referral network.

## Timor-Leste

### Building back after conflict

Kristof Wing et al. (716–722) describe the creation of a national eye-care service.

## Global

### An evidence-based approach

Jacqueline Ramke et al. (695–704) review the evidence for national universal eye health plans.

### Thwarting *C. trachomatis*

Sarity Dodson et al. (723–725) compare frameworks for interventions needed to eliminate trachoma in 57 countries.

### Population-based estimates of avoidable blindness

Islay Mactaggart et al. (726–728) outline methodological improvements to the rapid assessment of avoidable blindness.

